# Adherence to recommendations for secondary prevention medications after myocardial infarction in Estonia: comparison of real-world data from 2004 to 2005 and 2017 to 2018

**DOI:** 10.1186/s12872-021-02321-x

**Published:** 2021-10-20

**Authors:** Piret Lõiveke, Toomas Marandi, Tiia Ainla, Krista Fischer, Jaan Eha

**Affiliations:** 1 Department of Cardiology, University of Tartu, Tartu, Estonia; 2 Centre of Cardiology, North Estonia Medical Centre, 19 J. Sütiste Str, 13419 Tallinn, Estonia; 3 Quality Department, North Estonia Medical Centre, Tallinn, Estonia; 4 Institute of Mathematics and Statistics, University of Tartu, Tartu, Estonia; 5 Heart Clinic, Tartu University Hospital, Tartu, Estonia

**Keywords:** Myocardial infarction, Secondary prevention, Medication adherence, Guideline adherence

## Abstract

**Background:**

Relatively high rates of adherence to myocardial infarction (MI) secondary prevention medications have been reported, but register-based, objective real-world data is scarce. We aimed to analyse adherence to guideline-recommended medications for secondary prevention of MI in 2017 to 2018 (period II) and compare the results with data from 2004 to 2005 (period I) in Estonia.

**Methods:**

Study populations were formed based on data from the Estonian Health Insurance Fund’s database and on Estonian Myocardial Infarction Register. By linking to the Estonian Medical Prescription Centre database adherence to guideline-recommended medications for MI secondary prevention was assessed for 1 year follow-up period from the first hospitalization due to MI. Data was analysed using the defined daily dosages methodology.

**Results:**

Total of 6694 and 6060 cases of MI were reported in periods I and II, respectively. At least one prescription during the follow up period was found for beta-blockers in 81.0% and 83.5% (*p* = 0.001), for angiotensin converting enzyme inhibitor/angiotensin II receptor blocker (ACEi/ARB) in 76.9% and 66.0% (*p* < 0.001), and for statins in 44.0% and 67.0% (*p* < 0.001) of patients in period I and II, respectively. P2Y12 inhibitors were used by 76.4% of patients in period II. The logistic regression analysis adjusted to gender and age revealed that some drugs and drug combinations were not allocated similarly in different age and gender groups.

**Conclusions:**

In Estonia, adherence to MI secondary prevention guideline-recommended medications has improved. But as adherence is still not ideal more attention should be drawn to MI secondary prevention through systematic guideline implementation.

## Background

In recent years, a significant slowdown has occurred in the declining pace of CV mortality in high-income countries. It can, at least partly, be attributed to the rapidly growing populations of diabetic, obese and octogenarian people. It has been shown that nearly half of the considerable decline in CV mortality in the past decades can be explained by the use of evidence based medical treatment and interventions and approximately 44% by the reductions in major risk factors [1]. As shown by a body of evidence from research the potential of the guideline-recommended therapies has not been fully realized. So now, the key to further lowering CV mortality may lie in better adherence to secondary prevention medical treatment and risk factor control [2].

In Estonia, during the last decades, CV mortality has declined and reached a level of 198/100,000 in 2017 [3]. Highest CV mortality rate in the Western Europe was 158/100,000 observed in the 1970s [4, 5]. Assuming Estonia is following the CV mortality course of the western European countries with a time shift, a stagnation of the favourable trend is imminent.

Moderate to high rates of adherence to MI secondary prevention guideline-recommended medications have been reported in Europe by several different studies [6, 7] e.g. the EUROASPIRE V [8].

As could be expected, results vary according to the study method used. Some studies of MI secondary prevention guideline-recommended medication adherence analyse data of selected patient populations, often drawing the age limit at 75 or 80 years [8] or excluding patients for other reasons (medication not initiated at discharge, absence of certain insurance coverage, complications of treatment or disease, concomitant conditions) [6, 9]. Oftentimes, medication adherence is evaluated based on patient interviews [8] or targeting to only some recommended drug classes [9]. Therefore, registry based objective real-world data of secondary prevention medication adherence is scarce.

As mentioned, the standstill in the decline of CV mortality can partly be attributed to the ageing of the population. The elderly age group is becoming more prominent with problems of multiple comorbidities and frail overall condition posing new challenges to physicians and the whole society. So far, the > 80 years age group has often been excluded from the MI secondary prevention registries and studies, making objective information about secondary prevention medication adherence regarding the seniors, scarce.

In Estonia, collecting data of all MI patients in a comprehensive registry started in 2012. Ongoing data collection of an unselected patient population with linking data to the Estonian Causes of Death Registry and the Estonian Medical Prescription Center (EMPC) database on the individual level is a unique opportunity to evaluate treatment and outcome changes on a complete population cohort over time.

The aim of our study was to analyse adherence to guideline-recommended medications for secondary prevention of MI in 2017–2018 and compare the results with data from 2004–2005 in Estonia.

## Methods

Estonian Myocardial Infarction Register (EMIR) is a national ongoing register collecting data regarding all patients hospitalized with diagnosis of acute MI (International Classification of Diseases 10th version (ICD-10) codes I21-I22) from all Estonian hospitals with the purpose of national statistics and research. It is permitted by the Estonian law to use personal data from EMIR without informed consent of the participants for research purposes and linking with other national databases. EMIR was founded in 2012 and approximately 2700 cases are reported annually. Data reporting is mandatory by law. An electronic form comprising nearly 100 characteristics about patients’ CV risk factors, concomitant diseases, in-hospital treatment (procedures and medications) and recommended drug therapy at discharge is used. EMIR is linked annually with the Estonian Causes of Death Registry and Estonian Health Insurance Fund (EHIF) database by personal identification number.

EHIF is a national health insurance schema based on solidarity and equality meaning that working social taxpayers ensure equal quality and availability of health care services to all people covered by the insurance (working people, children and adolescences, elderly, pregnant women). In 2018 94.5% of the Estonian population was covered with the health insurance [10]. A database of all hospitalizations of insured patients is available. Data about prescribed and reimbursed medications is collected in the EMPC.

For adequate comparison of the results, this study was designed similarly to the one carried out in 2010 by Marandi et al*.* [11] using the defined daily dosages methodology. Briefly, for the earlier study the EHIF prepared a list of inpatients treated for MI (ICD-10 code I21-I22) during the period of 01.01.2004–31.12.2005 (period I). This list was then used to identify all prescriptions of statins (Anatomical Therapeutic Chemical Classification System (ATC) code C10AA), ACEis (ATC code C09AA), ARBs (ATC code C09CA) and BBs (ATC code C07A) reimbursed to these MI patients during a 12-month period (365 days) after the index episode. In the final analysis, data of patients who survived > 30 days from the index episode was used. Mortality data was obtained from the Estonian Population Registry for the 12-month period after the index episode. The index episode was defined as the first hospitalization due to MI during study period.

The present study was approved by the Research Ethics Committee of the University of Tartu. A list of patients was acquired from EMIR comprising all cases of MI (ICD-10 codes I21-I22) from 01.01.2017 to 31.12.2018 (period II). This list was linked to the EHIF’s database and the Estonian Causes of Death Registry. An index episode was defined as the first hospitalization of a person due to MI during the study period. Follow up time was defined as 365 days period after the index episode. Information regarding patients who lived > 30 days after the index episode was used in analysis of drug utilization. A list of medications of interest prescribed and reimbursed during the period of 183 days prior (i.e., patient had a valid prescription for medication of interest at the index episode time) and 365 days after the index episode was issued by the EMPC. Medications of interest were guideline recommended drugs for secondary prevention of MI [12]—statins, ACEis, ARBs, BBs, clopidogrel (ATC code B01AC04) and ticagrelor (ATC code B01AC24). All medications reimbursed were considered as used by the patient.

To analyse drug use for secondary prevention the daily defined dosages (DDD) methodology was used. It is a methodology developed by World Health Organisation (WHO): a defined daily dosage is the assumed average maintenance dose per day for a drug used for its main indication in adults. The DDDs were as follows: atorvastatin 20 mg, simvastatin 30 mg, fluvastatin 60 mg, rosuvastatin 10 mg, pravastatin 30 mg, ramipril 2.5 mg, enalapril 10 mg, perindopril 4 mg, fosinopril 15 mg, lisinopril 10 mg, trandolapril 2 mg, telmisartan 40 mg, losartan 50 mg, valsartan 80 mg, candesartan 8 mg, olmesartan 20 mg, carvedilol 37.5 mg, metoprolol 150 mg, propranolol 160 mg, sotalol 160 mg, atenolol 75 mg, nebivolol 5 mg, bisoprolol 10 mg, clopidogrel 75 mg, ticagrelor 180 mg [13]. As antiaggregants, only P2Y12 inhibitors clopidogrel and ticagrelor were studied in period II. In period I ticagrelor was not yet on the market and clopidogrel did not have a recommendation as a first-line choice to MI patients in Estonia. So data about P2Y12 inhibitors was not collected in period I. Acetylsalicylic acid is an over-the-counter drug and no reliable information for its utilization is available. Prasugrel is not reimbursed by the EHIF and was very rarely used some years ago in Estonia. DDDs were summarized based on drug class and divided by patient’s lifedays during follow-up.

As personalized data was used for linking purposes from all beforementioned registries (EMIR, EHIF, EMPC, Estonian Cause of Death Registry) the datasets are not openly accessible. An enquiry was made to the registries for accessing the data. Personal data from registries was pseudonymized and handled according to all personal data protection requirements.

### Statistical analysis

Categorical data were expressed as frequencies and compared with Pearson’s χ^2^ test. Age was presented as mean with standard deviation and compared between genders with the two-sample *t* test. The DDDs were summarized based on drug class and expressed as medians per life days by age groups. 95% confidence intervals for medians and α values for comparison between genders were obtained with Mann Whitney U test. Logistic regression models were fitted to explore associations between age, sex and allocated drugs and drug combinations. Results were expressed as odds ratios with 95% confidence intervals. Comparison of results between studies regarding periods I and II was done using frequency tables.

An α value of < 0.01 was considered significant. Analysis was performed with statistical software package RStudio version 1.2.5033 (RStudio, Boston, Massachusetts, USA).

## Results

Total of 6694 and 6060 cases of MI were reported and out of these 4900 and 5067 index episodes were defined in periods I and II, respectively. Mean age for men was 65.66 years (SD ± 11.56) and 67.5 years (SD $\pm \hspace{0.17em}$12.3) and for women 73.61 years (SD ± 9.81) and 77.4 years (SD $\pm \hspace{0.17em}$10.9) in period I and II, respectively. In comparison of period I *vs* II the 30-day and 1-year mortality decreased (Table 1). Also, the mean age for both gender groups rose over the past 13 years.

**Table 1:** Characteristics of study populations

	Period I (2004–2005)		Period II (2017–2018)		*p* value (comparison between periods)
	Men	Women	Men	Women	
Total number of index episodes, (%)	2772 (56.6)	2128 (43.4)*	3039 (60.0)	2028 (40.0)*	
One-year mortality, no. (%)	709 (25.6)	744 (35.0)*	581 (19.1)	602 (30.0)*	< 0.001
30-day mortality, no. (%)	407 (15.0)	468 (22.0)*	335 (11.0)	360 (17.8)*	< 0.001
*Study population*					
Number of patients who survived > 30 days, (%)	2365 (85.3)	1660 (78.0)*	2704 (89.0)	1668 (82.2)*	< 0.001
Age (years; mean, $\pm$ SD)	64.7 $\pm \hspace{0.17em}$11.5	72.7 $\pm \hspace{0.17em}$9.9*	66.5 $\pm \hspace{0.17em}$12.1	76.4 $\pm \hspace{0.17em}$10.9*	< 0.001
20–39 years, no. (%)	34 (1.4)	4 (0.2)	29 (1.1)	6 (0.4)	
40–59 years, no. (%)	740 (31.3)	166 (10.0)	779 (28.8)	126 (7.6)	
60–79 years, no. (%)	1383 (58.5)	1075 (64.8)	1498 (55.4)	835 (50.0)	
> 80 years, no. (%)	208 (8.8)	415 (25.0)	398 (14.7)	701 (42.0)	
One-year mortality, no. (%)	302 (12.8)	276 (16.6)*	246 (9.1)	242 (14.5)*	< 0.001

*p < 0.01 for comparison between genders. Age was compared with the *t* test, otherwise Pearson’s χ^2^ test was used

### Drug utilization

In period I 94.4% of patients who survived > 30 days were treated with at least one drug of the recommended classes (statins, BBs, ACEis/ARBs). In period II, out of the 4372 patients who survived > 30 days, 4009 (91.7%) were treated with at least one of the guideline recommended drugs for MI secondary prevention (statins, ACEis/ARBs, BBs or P2Y12 inhibitors).

**Fig. 1:**
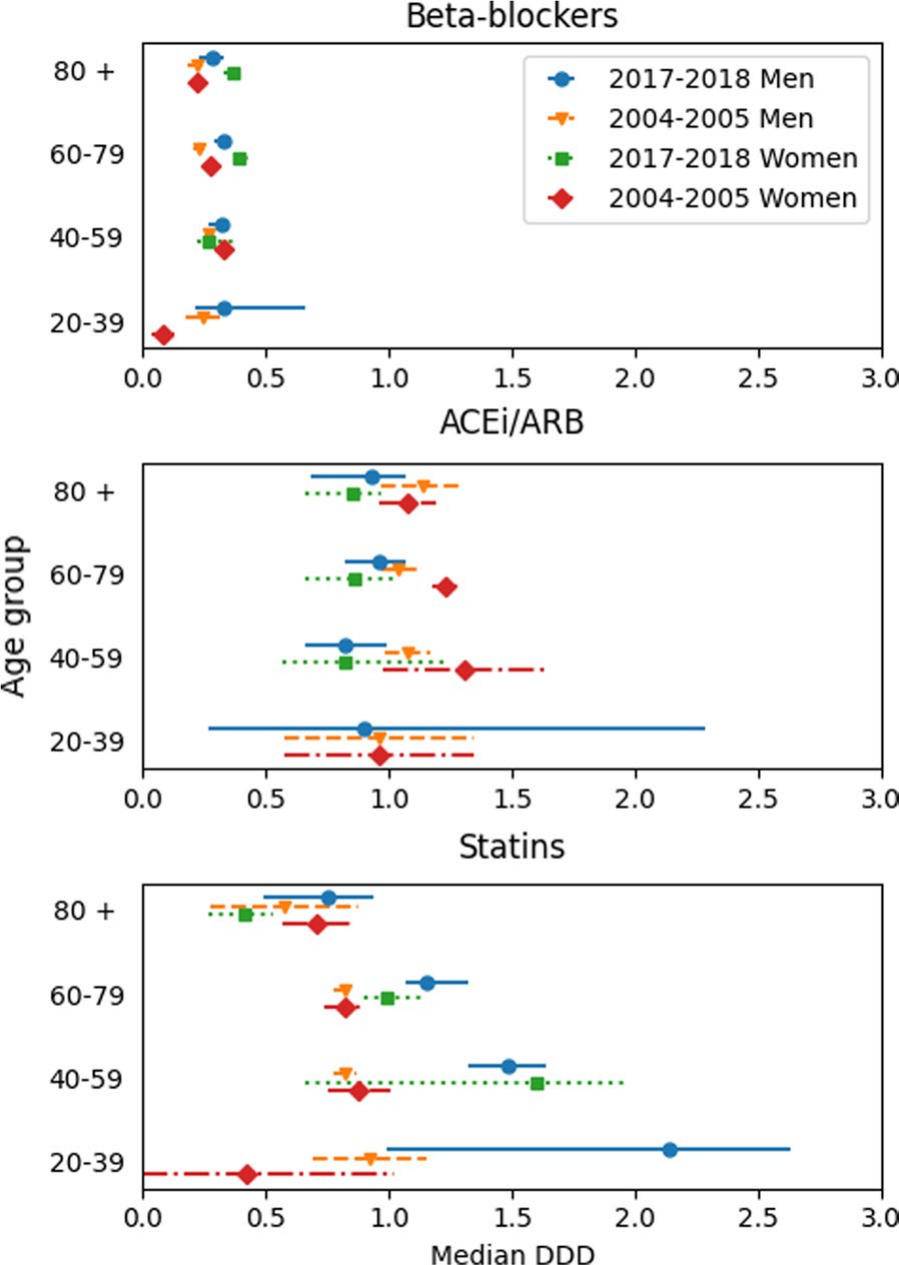
Median daily dosages of statins, angiotensin converting enzyme inhibitors/angiotensin II receptor blockers (ACEi/ARB) and betablockers (BB) presented as defined daily dosages (DDD) per lifeday with 95% confidence interval for age groups and gender for patients who survived > 30 days in comparison of periods I (2004–2005) and II (2017–2018)

In Fig. 1 median daily dosages of BBs, ACEis/ARBs and statins are presented as DDDs per lifeday with 95% confidence interval for age groups and gender in comparison of two study periods.

Median daily dosages for P2Y12 inhibitors were calculated only for period II and were consistently around 1.00 for all age and gender groups with the exception of the > 80 years age group. For this age group median daily dosages for P2Y12 inhibitors were 0.84 (95% CI 0.69–0.92) for men and 0.61 (95% CI interval 0.46–0.77) for women with a *p* = 0.0009.

With the exception of ACEi/ARB, representatives of all guideline-recommended drug classes were prescribed significantly more often in period II than in period I for patients who survived > 30 days after MI (Table 2).

**Table 2:** Proportion of patients with at least one prescription for guideline-recommended medications among patients who survived > 30 days

	Period I 2004–2005 (n = 4025)			Period II 2017–2018 (n = 4372)			*p* value (comparison between periods)
	Men	Women	Total	Men	Women	Total	
BB, no. (%)	1907 (80.6)	1344 (81.0)	3251 (81.0)	2265 (84.0)	1385 (83.0)	3650 (83.5)	0.001
ACEi/ARB, no. (%)	1780 (75.3)	1317 (79.3)	3097 (76.9)	1817 (67.2)	1070 (64.1)	2887 (66.0)	< 0.001
Statins, no. (%)	946 (40.0)	826* (50.0)	1772 (44.0)	1910 (70.6)	1020* (61.2)	2930 (67.0)	< 0.001
P2Y12 inhibitors, no. (%)	*NA*	*NA*	*NA*	2194 (81.1)	1147* (69.0)	3341 (76.4)	*NA*

Pearson’s χ^2^ test used for comparison between periods

*BB* beta-blockers, *ACEi/ARB* angiotensin converting enzyme inhibitors/angiotensin II receptor blockers, *NA* not available

*P < 0.01 for comparison between men and women with Pearson’s χ^2^ test

Combinations of prescribed treatments for patients who survived > 30 days for the two study periods are presented in Table 3.

**Table 3:** Combinations of prescribed treatments for patients who survived > 30 days

	Period I 2004–2005 (n = 4025)		Period II 2017–2018 (n = 4372)		*p* value (comparison between periods)
	Men	Women	Men	Women	
Only BB, no. (%)	176 (7.4)	123 (7.4)	158 (5.8)	141* (8.5)	0.314
Only ACEi/ARB, no. (%)	170 (7.2)	145 (8.3)	39 (1.4)	31 (1.9)	< 0.001
Only statin, no. (%)	17 (0.7)	12 (0.7)	37 (1.3)	25 (1.5)	0.003
BB + ACEi/ARB, no. (%)	602 (25.5)	525* (31.6)	338 (12.5)	301* (18.0)	< 0.001
BB + statins, no. (%)	130 (5.5)	49* (3.0)	433 (16.0)	257 (15.4)	< 0.001
ACEi/ARB + statin, no. (%)	141 (6.0)	63* (3.8)	104 (3.8)	52 (3.1)	0.001
BB + ACEi/ARB + statin, no. (%)†	999 (42.2)	647 (39.0)	1336 (49.4)	686* (41.1)	< 0.001
None of the above medications, no. (%)	130 (5.5)	96 (6.0)	214 (7.9)	148 (9.0)	< 0.001

P2Y12 inhibitor use not accounted for. Pearson’s χ^2^ test used for comparison between periods

*BB* beta-blockers, *ACEi/ARB* angiotensin converting enzyme inhibitors/angiotensin II receptor blockers, *NA* not available

*P < 0.01 for comparison between men and women with Pearson’s χ^2^ test

Frequency of prescribing the triple combination of BBs, ACEis/ARBs and statins had increased (in total 40.8% in period I vs 46.2% in period II, *p* < 0.01) and a difference between genders had appeared.

**Fig. 2:**
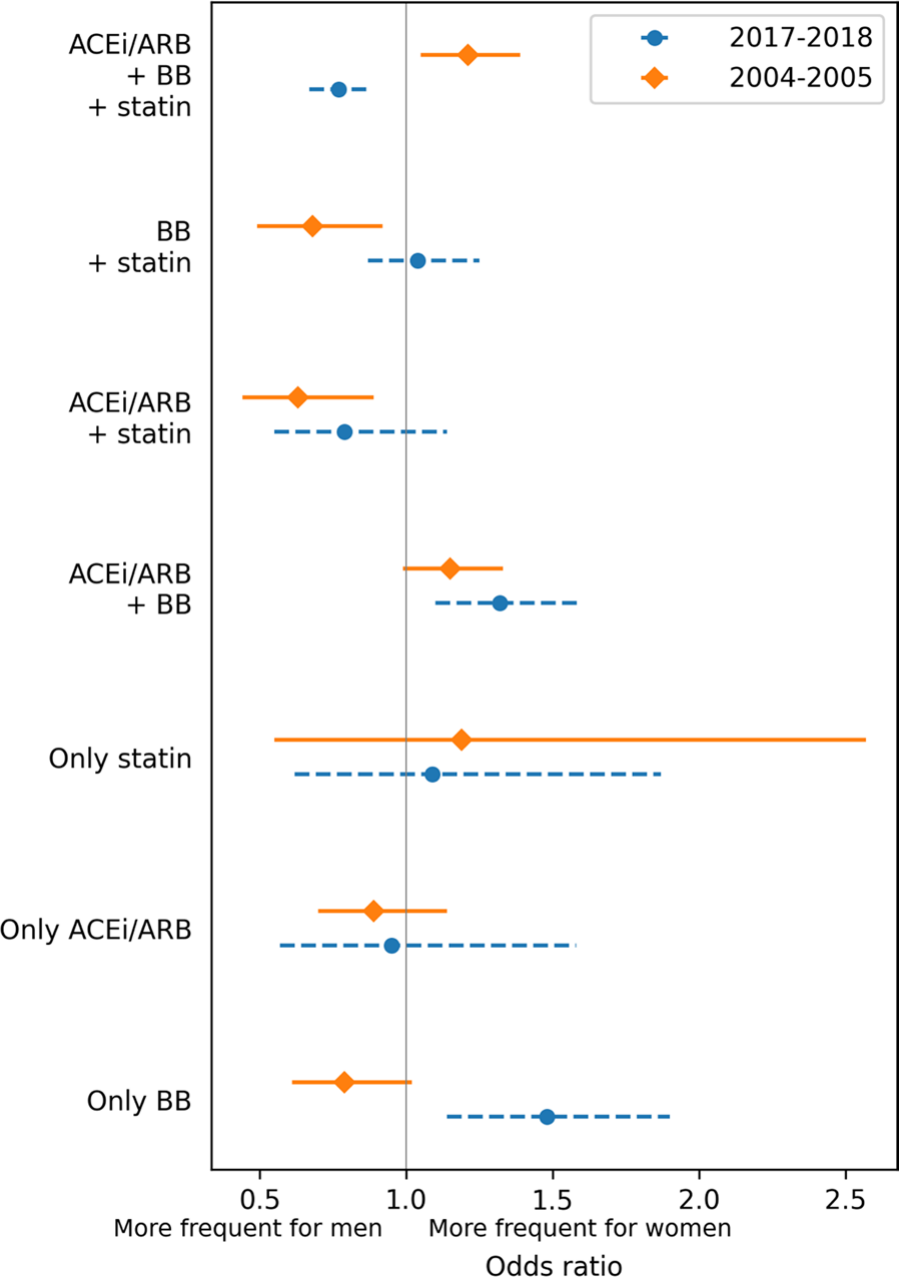
Relationship of drug allocation and gender in patients who survived > 30 days. *BB* beta-blockers, *ACEi/ARB* angiotensin converting enzyme inhibitors/angiotensin II receptor blockers, *OR* odds ratio. Men were used as reference groups for logistic regression model

In Fig. 2 results of logistic regression analysis for associations of drug allocation and gender for periods I and II are presented. Prescriptions for P2Y12 inhibitors were analysed only for period II. It was found that women were prescribed P2Y12 inhibitors significantly less often (OR 0.61, 95% CI 0.51–0.73, *p* < 0.001).

Results of logistic regression for drug allocation and age groups in both study periods are presented in Table 4.

**Table 4:** Relationship of drug allocation and age in patients who survived > 30 days in 2004–2005 (period I) and 2017–2018 (period II)

	20–59 years; OR (95% CI)	60–79 years; OR (95% CI)		> 80 years; OR (95% CI)	
		2004–2005	2017–2018	2004–2005	2017–2018
BB	1.00	0.97 (0.29–3.19)	1.00 (0.73–1.39)	1.93 (0.58–6.47)	1.08 (0.75–1.58)
ACEi/ARB	1.00	2.49* (1.67–3.70)	1.38 (0.67–3.12)	5.69* (3.66–8.82)	3.16** (1.49–7.35)
Statins	1.00	0.88 (0.38–2.06)	1.32 (0.68–2.75)	0.17 (0.02–1.37)	1.45 (0.50–2.69)
ACEi/ARB + BB	1.00	1.21 (0.57–2.57)	1.06 (0.84–1.36)	1.92 (0.89–4.14)	1.85** (1.42–2.42)
ACEi/ARB + statin	1.00	0.32* (0.12–0.84)	1.06 (0.7–1.72)	0.19* (0.06–0.58)	1.09 (0.66–1.81)
Statin + BB	1.00	0.81 (0.24–2.66)	1.01 (0.82–1.25)	0.28 (0.08–1.06)	0.75* (0.57–0.97)
BB + ACEi/ARB + statin	1.00	1.02 (0.53–1.96)	0.93 (0.79–1.09)	0.28* (0.14–0.55)	0.72** (0.59–0.87)
None of the above	1.00	NA	1.31 (1.00–1.73)	NA	1.66** (1.20–1.30)
BB + ACEi/ARB + statin + P2Y12 inhibitor	1.00	NA	0.82* (0.70–0.94)	NA	0.52** (0.42–0.63)
P2Y12 inhibitor	1.00	NA	1.31 (1.00–1.73)	NA	1.66** (1.20–1.30)

Due to small number of patients in 20–39 years and 40–59 years age groups they were merged and used as reference groups for logistic regression model

*BB* beta-blockers, *ACEi/ARB* angiotensin converting enzyme inhibitors/angiotensin II receptor blockers, *OR* odds ratio, *CI* confidence interval

*p < 0.05; **p < 0.001 for comparison between age groups within one time period

It can be seen that still the triple combination of BBs, ACEis/ARBs and statins was prescribed less often to older patients. Also, a significant difference between age groups was observed for P2Y12 inhibitors in period II (60–79 years OR 0.48, 95% CI 0.35–0.65, *p* < 0.001: > 80 years OR 0.18, 95% CI 0.13–0.24, *p* < 0.001).

## Discussion

Our study found that guideline-recommended medications for MI secondary prevention were prescribed more often in period II than in period I but there are significant differences among age and gender subgroups. BBs are still prescribed in smaller dosages than recommended in guidelines. In addition, we found that during the past 13 years the mean age of MI patients has increased but the 30-day and 1-year mortality of MI patients in Estonia decreased significantly.

A positive shift has taken place in prescribing guideline recommended MI secondary prevention medications in Estonia, but our patients’ rate of adherence is still lower than in several studies conducted elsewhere in Europe. The EUROASPIRE V study [8] looking into secondary prevention practices in 27 European countries reported 81% of patients receiving BBs, 75% receiving ACEis or ARBs and 80% receiving statins after > 6 months from MI. 93% of patients received anti-platelets in EUROASPIRE V, but aspirin, besides P2Y12 inhibitors, was also counted for. It is of importance, that only patients < 80 years of age were included in EUROASPIRE V and data was derived from patient interviews. Gross et al. [6] also reported high adherence to MI secondary prevention medications in an intervention program with regular visits to office-based cardiologist in Germany, proving that in a selected group of highly motivated patients who are willing to participate in high intensity secondary prevention programs, good results are achievable. But in every day practice, as did also our data show, on unselected patient populations the results are far from ideal with moderate rates of adherence also reported by Huber et al*.* in Switzerland [7].

In our study an unselected population of MI patients was observed, and it was evident that women and the elderly (patients in > 80 years age group) were considerably less likely to receive statins and P2Y12 inhibitors. Saar et al*.* [14] showed that in Estonia, elderly patients, who comprise nearly 80% of the GRACE score high risk population, undergo percutaneous coronary intervention (PCI) and use P2Y12 inhibitors significantly less often during hospitalization. Also, a correlation between performing PCI and use of P2Y12 inhibitors in other age groups was demonstrated.

In addition, we found that the triple combination of BBs, ACEis/ARBs and statins was significantly less often prescribed to women and the elderly. Again, these findings correlate to other studies on unselected population [7] and could partly explain lower rates of adherence in our study compared to results from selected patient populations.

It is common practice that physicians are cautious when prescribing secondary prevention medications to elderly patients with the concerns of possible adverse effects, drug-drug interactions and questionable benefit in mind [15–17]. Recently evidence has shown that elderly patients do benefit from guideline recommended secondary prevention drug therapy after MI without any substantial additional harm [18–21]. The demographic group of elderly MI patients needs to be highlighted and large-scale registry based studies conducted to find opportunities for improvement in the challenging task of secondary prevention medical therapy in seniors.

Also, the fact that women use less guideline recommended MI secondary prevention drug treatment is a recognized phenomenon [16, 22, 23]. In our study cohorts women constituted the majority in the > 80 years age group. The seniors are more likely to be burdened with several comorbidities and bare the higher risk of treatment complications and drug side-effects which all may contribute to the reluctance to prescribe medication to these patients and partly explain the lower adherence rate among women. It has also been proposed that women may receive less guideline recommended secondary prevention medications due to the perception that medications are less efficacious in women or have more pronounced side-effects [24]. For instance, women are shown to have more often non obstructive coronary disease compared to men which may lead physicians to withhold medications like statins and aspirin, used primarily for obstructive coronary artery disease [22].

Rate of prescribing BBs has increased significantly over time in Estonia. However, regardless of sex and age, beta blocking agents were used in relatively small dosages—an issue also evident in 2004. It is a universally recognized problem which still is not very well explained [25]. At one hand, the length of hospital stay is increasingly shortening and follow up visits are infrequent which discourage general practitioners from up titrating the initial dose. On the other hand, evidence form research has risen the question of appropriateness of the guideline recommended doses [26] and indication of BBs in the PCI, antiplatelet and statin era [27].

The number of patients to whom no secondary prevention medications were prescribed, had risen from 5.6% in period I to 8.3% in period II. The majority of these patients were in the > 60 years age groups, for 42.4% of these patients no coronary angiography was performed. 18.5% of them died during the follow up in the period II cohort. No description for the period I cohort was available. The reasons for this slight negative tendency can only be assumed (*e.g.,* more patients survive out of hospital cardiac arrest who remain with a profound cognitive deficit and are managed in nursing homes, some patients leave the country etc*.*) and need to be investigated further.

In Estonia, the CV disease mortality rate is declining. The MI in hospital management has improved considerably [28] as can be concluded from the decreased 30-day mortality rate. But as the number of diabetics and overweight patients, often already in younger age groups, and octogenarians is increasing, solutions for achieving and maintaining MI secondary prevention guideline recommended goals (including high adherence to medical therapy), are needed [29].

### Strengths and limitations

The major strength of this study is the characteristics of the data—full representation of the MI patients’ population without a selection bias. Also, information is available without interviewer bias or recall bias.

Our study has certain limitations. Firstly, the defined daily dosages methodology used is not the most accurate and probably overestimates adherence slightly. It was chosen, instead of medication possession rate methodology, to enable comparison with the previously conducted study using the DDD methodology. By using this methodology, we cannot differentiate everyday-users from short-time-users of certain drug.

Secondly, there is no data available to confirm whether the patients actually take the purchased medications. So, the actual usage is probably lower than the presented results. But still, accounting reimbursed prescriptions as used medications by patient is a validated methodology [30].

Thirdly, we do not have any information to explain the reasons behind nonadherence nor for not finding any prescriptions in the database for some patients.

Fourthly, we have no data about contraindications or information about intolerance of recommended dosages regarding individual patients which may explain some of the nonadherence to guidelines.

Finally, we had no information regarding comorbidities nor other socioeconomic factors other than age and sex available for the 2004–2005 cohort. To follow the methodology used by Marandi et al. [11] in the earlier study, this information was not analyzed for the 2017–2018 cohort, either. Probably, this background information would have helped to explain the adherence to some extent.

## Conclusions

In Estonia, adherence to MI secondary prevention guideline-recommended medications has improved over 13 years. According to our results, adherence is still not ideal, more attention should be drawn especially to women and the elderly. A systematic approach for delivering MI guideline-recommended secondary prevention should be implemented in Estonia, to reduce the CV mortality further.

## Data Availability

Due to the lack of a publicly accessible data repository, the datasets used and/or analysed during the current study are available from the corresponding author on reasonable request.

## Abbreviations

ACEi: Angiotensin converting enzyme inhibitor
ARB: Angiotensin receptor antagonist
ATC: Anatomical Therapeutic Chemical Classification System
BB: Beta blocker
CI: Confidence interval
CV: Cardiovascular
DDD: Daily defined dosages
EHIF: Estonian Health Insurance Fund
EMIR: Estonian Myocardial Infarction Register
EMPC: Estonian Medical Prescription Center
EUROASPIRE: European Action on Secondary and Primary Prevention by Intervention to Reduce Events
GRACE: Score the Global Registry of Acute Coronary Events score
ICD-10: International Classification of Diseases 10th version
MI: Myocardial infarction
OR: Odds ration
PCI: Percutaneous coronary intervention
SD: Standard deviation
WHO: World Health Organisation
